# Adolescent Sports Learning Interest and Subjective Well-Being: The Chain Mediating Role of Social Anxiety and Sleep Quality

**DOI:** 10.3390/bs16050721

**Published:** 2026-05-07

**Authors:** Hanlin Qi, Natchana Bhutasang, Shixiang Liu, Wen Zhang

**Affiliations:** 1 Faculty of Psychology, Shinawatra University, Bang Toey, Sam Khok, Pathum Thani 12160, Thailand; 67427007-9@st.siu.ac.th (H.Q.); natchana.b@siu.ac.th (N.B.); 2 Department of Psychology, Teachers College of Beijing Union University, Beijing 100011, China; liusx@buu.edu.cn; 3 School of Psychology, Qufu Normal University, No. 57 Jingxuan West Road, Qufu 273165, China

**Keywords:** adolescent, sports learning interest, subjective well-being, social anxiety, sleep quality, chain mediation

## Abstract

Objective: While the benefits of physical activity are well-documented, the internal mechanisms linking sports interest to adolescent Subjective Well-being (SWB) remain under-explored. This study addresses this gap by investigating the association between Sports Learning Interest and SWB and examining the serial mediating roles of Social Anxiety and Sleep Quality. Methods: Using a stratified random sampling method, 1764 primary and secondary students were surveyed with validated instruments, including the PSQI and the Index of Well-Being. Data were analyzed using Hayes’ PROCESS macro (Model 6) to test the hypothesized chain mediation effects. Results: Sports Learning Interest significantly and positively predicted SWB. The model identified three significant indirect pathways: independent mediation by Social Anxiety, independent mediation by Sleep Quality, and a serial chain from Social Anxiety → Sleep Quality (total indirect effect = 0.18, 95% CI [0.15, 0.21]). Notably, Sleep Quality emerged as the core mediator, accounting for 29.9% of the total effect. Conclusions: Enhancing sports interest is positively associated with SWB through a “psychological-physiological” dual channel—specifically by its association with lower social anxiety and subsequently better sleep. These findings provide empirical evidence for designing school-based interventions that integrate physical education with mental health promotion.

## 1. Introduction

### 1.1. Research Background and Theoretical Basis

In the contemporary global educational context, adolescent mental health has emerged as a critical challenge within the domain of public health. As academic pressures intensify and social environments become increasingly complex, the psychological resilience of youth is continuously tested, with rising prevalence rates of anxiety, depression, and sleep-related issues reported globally (Cheng et al., 2025). Subjective Well-being (SWB), serving as a core indicator of an individual’s quality of life and psychological adaptation, encompasses not only an overall cognitive evaluation of life satisfaction but also the continuous experience of positive affect and the effective regulation of negative affect (Diener, 1984; Campbell et al., 1976). It is a multifaceted construct that reflects an individual’s optimal psychological functioning.

Prior research has extensively confirmed that Physical Activity (PA) is a vital instrument for enhancing SWB (Chen & Cheng, 2025; McMahon et al., 2017). The mechanisms underlying this relationship are often attributed to the release of endorphins, the reduction in cortisol levels, and the enhancement of self-efficacy. Furthermore, the efficacy of PA in improving Sleep Quality is supported by systematic reviews and significant physiological and psychological mechanisms (Alnawwar et al., 2023; J. X. Wang et al., 2026). This intervention effect has gained widespread recognition in clinical nursing and public health fields. Specifically, moderate-intensity aerobic exercise is of significant value in shortening sleep latency and increasing slow-wave sleep (Han et al., 2025). Furthermore, a decline in Sleep Quality affects not only emotion regulation but has also been confirmed as an independent predictor of impaired physical function, such as an increased risk of sports injuries (Watson et al., 2020). Thus, the interplay between PA, sleep, and mental health forms a crucial triad for adolescent development.

However, existing literature often possesses limitations by predominantly focusing on extrinsic behavioral indicators, such as exercise load, frequency, or duration, while neglecting the intrinsic psychological dynamics that support behavioral persistence. Self-Determination Theory (SDT) posits that individual behavioral motivation exists on a continuum from controlled to autonomous, wherein interest serves as the most direct manifestation of Intrinsic Motivation (Ryan & Deci, 2017). Unlike forced exercise, which may induce stress, interest-driven activity fosters a sense of volition. Research indicates that a positive peer atmosphere, by supporting intrinsic motivation, can effectively predict an individual’s need satisfaction and behavioral persistence in sports (Jõesaar et al., 2011). When students exhibit a profound interest in sports learning—termed here as Sports Learning Interest—the satisfaction of their basic psychological needs (Autonomy, Competence, and Relatedness) is viewed as a fundamental driver promoting well-being. Evidence from systematic reviews and intervention research indicates that PA, by satisfying individuals’ needs for autonomy, competence, and relatedness, is significantly associated with their SWB (Iwon et al., 2021; R. Li & Huang, 2025).

Consequently, exploring how Sports Learning Interest relates to SWB by enhancing psychosocial adaptation (reducing Social Anxiety) and physiological functional restoration (improving Sleep Quality) holds significant theoretical value and practical significance for constructing a comprehensive mental health promotion system for adolescents. This study aims to move beyond simple correlation analyses to unpack the “black box” of mechanisms linking interest to well-being via a chain mediation model.

### 1.2. The Relationship Between Sports Learning Interest and SWB

Sports Learning Interest is not merely a precipitating factor for learning behavior; it is a vital psychological resource for the individual. According to the Broaden-and-Build Theory of positive emotions, interest acts as a positive emotional trait that can broaden an individual’s momentary thought–action repertoires. Unlike negative emotions that narrow focus (e.g., fight or flight), interest encourages exploration, play, and integration. This broadening effect builds enduring psychological, physical, and social resources (Fredrickson, 2001).

Students with high Sports Learning Interest are more likely to enter a state of “Flow” during sports participation—a state of complete absorption where self-consciousness vanishes and time distorts. This deep immersion generates immediate positive affect. A large-scale meta-analysis regarding leisure sports confirmed a robust positive correlation between interest-driven PA and SWB (Wiese et al., 2018). Empirical studies show that emotional experiences during exercise—such as sport enjoyment—are core drivers promoting sustained sports participation and positively relating to life satisfaction among adolescents (Peng et al., 2024). Furthermore, physical exercise does not merely elevate well-being directly but also achieves this goal by reinforcing the individual’s positive core schemas, such as self-competence and physical self-concept (W. P. Zhang et al., 2025). When students feel competent and interested in sports, they develop a positive self-identity that permeates other life domains, which is closely associated with overall SWB.

### 1.3. The Mediating Role of Social Anxiety

Social Anxiety is a prevalent psychological distress among adolescents, characterized by a marked fear of social situations in which the individual is exposed to possible scrutiny by others. Based on the perspective of relatedness needs within SDT, the physical education (PE) classroom provides a natural, non-threatening, and informal social laboratory. High Sports Learning Interest drives students to actively seek peer interaction during team competitions or collaborative learning tasks. This interest-based behavioral engagement can generate a “natural desensitization” effect. Through repeated, interest-driven exposure to social interactions in sports (e.g., passing a ball and coordinating a defense), individuals gradually desensitize to the fear of negative evaluation and exhibit stronger social resilience (Eime et al., 2013).

Longitudinal tracking studies further confirm that sports participation significantly predicts a reduction in social anxiety levels in later adolescence (Brière et al., 2018). Participation in sports fosters a sense of belonging and provides a structured environment for social skill acquisition. Since Social Anxiety is a strong negative correlate of SWB—as it leads to isolation and loneliness—a reduction in its level may directly correspond to an improvement in an individual’s interpersonal well-being. This effect is particularly evident in sports contexts, as healthy peer attachment relationships play a key mediating role between physical exercise and well-being (W. P. Zhang et al., 2025).

### 1.4. The Mediating Role of Sleep Quality

Sleep Quality is a critical foundational pillar of adolescent physical and mental health. Systematic reviews focusing on adolescents clearly point out that sleep duration and quality are robust indicators predicting positive emotion and life satisfaction (Short et al., 2020). The optimization of sleep by Sports Learning Interest can be summarized as a “psychosomatic synergistic pathway.”

In the physiological dimension, interest-driven regular exercise helps regulate the body’s circadian rhythms and homeostatic sleep drive. It regulates the balance between excitation and inhibition in the central nervous system, promoting deeper, more restorative sleep. In the psychological dimension, interest serves as a buffer against stress, reducing the individual’s daytime anxiety levels which often interfere with sleep onset. High-quality sleep maintains internal homeostasis and enhances the prefrontal cortex’s regulation of emotional responses (Gruber et al., 2012), thereby positively relating to the individual’s SWB.

It is crucial to distinguish between sleep duration and quality. Compared to mere sleep duration, Sleep Quality (encompassing latency, efficiency, and disturbances) is more closely related to adolescent SWB and emotional fluctuations (Shen et al., 2018). A study based on Ecological Momentary Assessment (EMA) tracked individuals’ daily lives in real time and found that sleep satisfaction (rather than just sleep duration) is a robust predictor of next-day SWB (including positive affect and life satisfaction) (Lenneis et al., 2024).

### 1.5. The Chain Mediating Path of Social Anxiety and Sleep Quality

Beyond independent mediation, there is strong theoretical reason to suspect a causal chain between social anxiety and sleep. According to the Cognitive Model of Insomnia (Harvey, 2002), cognitive arousal triggered by Social Anxiety is a key antecedent disrupting sleep. Individuals with high Social Anxiety often engage in post-event processing or “social rumination” before sleep—replaying daytime social interactions, analyzing perceived mistakes, and worrying about future evaluations. This cognitive “hyperarousal” activates the sympathetic nervous system, interferes with sleep initiation (increasing latency), and degrades Sleep Quality (fragmenting sleep).

Sports Learning Interest, by improving the individual’s daytime interpersonal adaptation and providing positive social experiences, can effectively interrupt this negative cognitive feedback loop. If a student experiences success and enjoyment in sports interactions during the day, their pre-sleep cognitions are less likely to be dominated by anxious rumination. This forms a virtuous cycle of “Comfortable Daytime Interaction—Peaceful Nighttime Rest—Long-term Happiness.”

### 1.6. Research Gap

While the aforementioned literature highlights the benefits of PA, a distinct research gap remains. First, previous studies have largely prioritized extrinsic PA metrics, leaving the internal mechanism of intrinsic motivation—such as Sports Learning Interest—under-explored in the context of adolescent well-being. Second, although the independent correlations of PA with social anxiety and sleep quality are established, the specific, sequential internal mechanisms linking sports interest to adolescent SWB have not been adequately modeled. Specifically, whether social anxiety and sleep quality operate sequentially as a chain mediation process remains a missing link in current educational and psychological research. Addressing these gaps is crucial to providing empirical evidence for designing school-based interventions that integrate physical education with mental health promotion through intrinsic motivation.

### 1.7. Research Hypotheses

To address the identified research gaps, this study proposes a chain mediation model to examine the following hypotheses:

**H1.**  *Sports Learning Interest directly and positively predicts the SWB of adolescent students.*

**H2.**  *Social Anxiety plays a mediating role between Sports Learning Interest and SWB.*

**H3.**  *Sleep Quality plays a mediating role between Sports Learning Interest and SWB.*

**H4.**  *Social Anxiety and Sleep Quality play a chain mediating role in the pathway through which Sports Learning Interest is associated with SWB.*

## 2. Materials and Methods

### 2.1. Participants

This study employed a stratified random sampling method to ensure that the sample was representative of the adolescent population. A total of 1904 students were surveyed from six primary and secondary schools, covering three educational stages: elementary (grades 4–6), middle (grades 7–9), and high schools (grades 10–12). The gender distribution was approximately 54% male and 46% female. With the consent of the schools and the students’ guardians, questionnaires were distributed for the survey. Stringent data-cleaning procedures were applied; 142 questionnaires that showed tendencies of random responding (e.g., straight-lining) or had significant missing values were excluded. The final valid sample consisted of 1764 students, providing sufficient statistical power for complex mediation modeling.

### 2.2. Measurement Tools

#### 2.2.1. SWB

The Index of Well-Being developed by Campbell et al. (1976) was utilized to assess SWB. This instrument comprises two distinct sections: the “Index of General Affect” scale (8 items, weight = 1) and the “Life Satisfaction” questionnaire (1 item, weight = 1.1). The General Affect items assess the frequency of various emotions, while the Life Satisfaction item provides a global assessment. The scale utilizes a 7-point scoring system, with scores ranging from 2.1 (least happy) to 14.7 (happiest). The weighted scoring method allows for a balanced view of both affective and cognitive components of well-being. Previous research has demonstrated that the reliability and validity of this scale meet statistical requirements in Chinese populations (X. D. Wang et al., 1999). In the present study, the Cronbach’s α coefficient for this scale was 0.93, a result higher than in previous studies, indicating the scale possesses highly satisfactory reliability.

#### 2.2.2. Sleep Quality

The Pittsburgh Sleep Quality Index (PSQI), developed by Buysse et al. (1989), was employed (19 items). The scale assesses the subject’s sleep status over the past month and consists of 7 components:

1. Subjective sleep quality;
2. Sleep latency (time taken to fall asleep);
3. Sleep duration;
4. Sleep efficiency;
5. Sleep disturbances (e.g., waking up, pain);
6. Use of sleeping medication;
7. Daytime dysfunction (e.g., staying awake while driving/eating).

Each component is scored on a scale of 0–3, and the scores of the components are summed to yield the PSQI total score. The PSQI total score ranges from 0 to 21, where higher scores indicate poorer Sleep Quality. Existing studies indicate that its reliability and validity meet psychometric requirements (Dai, 2010; Lu et al., 2014; Shen et al., 2018). In this study, the Cronbach’s α coefficient was 0.68; notably, after excluding the “sleeping medication” component (which had extremely low variance in this adolescent sample), the Cronbach’s α coefficient rose to 0.70, consistent with prior findings (Y. Wang et al., 2019).

#### 2.2.3. Social Anxiety

The Social Anxiety Scale, revised by Scheier and Carver (1985), was used. This scale is often a subscale of the Self-Consciousness Scale (SCS-R) or derived from Fenigstein et al. (1975), specifically measuring the anxiety related to being scrutinized in social settings. This scale employs a 4-point scoring system (0 = “not at all like me” to 3 = “a lot like me”). Its internal consistency reliability (Cronbach’s α) was reported as 0.79 in the previous literature (X. D. Wang et al., 1999). In the application of this study among primary and secondary school students, the Cronbach’s α coefficient was 0.74, indicating acceptable internal consistency for group comparisons.

#### 2.2.4. Sports Learning Interest

Although various measurement tools have been developed to assess sports learning interest (Chai & Lin, 2017), the Physical Education Learning Interest Scale for Primary and Secondary School Students (J. B. Lin & Chai, 2017) was adopted in this study. This scale was specifically designed for the Chinese educational context. It includes four factors. Based on the specific item content and the perspective of sports educational psychology, this study further clarifies the naming of these factors as follows:

1. Situational Interest Triggering: The initial stimulation of interest within the PE class context.
2. Situational Interest Maintenance: The ability to sustain interest during PE class activities.
3. Individual Interest Sprouting: The extension of sports learning interest to contexts outside of class (e.g., extracurricular sports).
4. Individual Interest Maturation: The internalization of sports interest into the self-concept.

Existing research indicates that the scale’s internal consistency reliability, split-half reliability, and test–retest reliability are 0.944, 0.872, and 0.813 respectively, with subscale reliabilities all above 0.70. In this study, the Cronbach’s α coefficient for the scale was 0.93, indicating excellent internal consistency.

### 2.3. Ethical Approval

This study was conducted in accordance with the Declaration of Helsinki and approved by the Ethics Committee of Shinawatra University (protocol code 027/2025 and date of approval: 5 September 2025).

This study was conducted in accordance with ethical standards. Informed consent was obtained from school administrators, parents, and students. Participation was voluntary, and anonymity was guaranteed to reduce social desirability bias.

### 2.4. Statistical Analysis

All statistical analyses were performed using IBM SPSS Statistics for Windows (Version 27.0) and Mplus 8.0 software.

1. Measurement Model Validation and Common Method Bias Test: Confirmatory Factor Analysis (CFA) was first conducted using Mplus to verify the factorial validity of the measurement models, particularly addressing the multidimensionality of the applied scales. Model adequacy was evaluated using standard fit indices (e.g., CFI, TLI, RMSEA, SRMR). Following the confirmation of construct validity, Harman’s single-factor test was performed to assess the presence of significant common method bias.
2. Descriptive Statistics: Means, standard deviations, and Pearson correlation coefficients were calculated using SPSS (Version 27.0) to examine the preliminary bivariate relationships between variables.
3. Mediation Analysis: Based on the validated measurement models, the PROCESS macro developed by Hayes (SPSS version, Model 6) was utilized to test the complex mediation model containing multiple mediators in a serial chain (Hayes, 2013). Bootstrapping with 5000 resamples was used to generate 95% confidence intervals (CI) for the conditional indirect effects; if the CI does not include zero, the effect is considered statistically significant.

## 3. Results

### 3.1. Measurement Model Validation and Common Method Bias Test

Prior to hypothesis testing, a Confirmatory Factor Analysis (CFA) was conducted using Mplus to verify the factorial validity of the measurement models. Specifically, Sports Learning Interest was modeled as a second-order factor alongside first-order factors of Social Anxiety, Sleep Quality, and SWB. The CFA results demonstrated the following fit indices: χ^2^ = 5801.177, *df* = 848, *p* < 0.001; RMSEA = 0.058 (90% CI: 0.056–0.059); SRMR = 0.067; CFI = 0.899; and TLI = 0.892. Although the CFI and TLI values are marginally below the traditional 0.90 threshold, methodological literature highlights that for complex measurement models with a large number of items, values approaching 0.90, coupled with excellent RMSEA (<0.06) and SRMR (<0.08), denote a highly acceptable model fit (Marsh et al., 2004). Furthermore, all standardized factor loadings for the indicators on their respective latent variables were statistically significant. Consequently, the factorial validity of the constructs was established.

Since the data in this study were obtained via self-report questionnaires, they may be subject to common method bias (CMB). To mitigate this, procedural remedies such as anonymous responses and reverse scoring were implemented during data collection. To statistically test for severe common method bias, Harman’s single-factor test was conducted using Confirmatory Factor Analysis (CFA). A single-factor model was constructed by loading all measurement indicators onto a single latent variable (Podsakoff et al., 2003). The results showed that the fit of the single-factor model was extremely poor (χ^2^/*df* = 26.95, RMSEA = 0.12, TLI = 0.42, and CFI = 0.44), far below conventionally accepted fit standards (e.g., RMSEA < 0.08 and CFI > 0.90). Therefore, it can be concluded that the variations in the data cannot be explained by a single common method factor, and no serious common method bias exists in this study.

### 3.2. Descriptive Statistics and Analysis of Main Variables

Table 1 presents the means, standard deviations, and correlation coefficients of the main variables. The results indicate significant correlations among all main variables in the expected directions:

- PSQI Total Score (Sleep Quality) was negatively correlated with Sports Learning Interest (*r* = −0.30, *p* < 0.01), and positively correlated with Social Anxiety (*r* = 0.34, *p* < 0.01). Note: Higher PSQI scores indicate worse sleep quality; thus, the negative correlation with Interest/SWB indicates that higher interest and well-being are associated with better sleep (lower PSQI scores).
- Sports Learning Interest was negatively correlated with Social Anxiety (*r* = −0.30, *p* < 0.01) and positively correlated with SWB (*r* = 0.35, *p* < 0.01). This provides preliminary support for H1.
- Social Anxiety was negatively correlated with SWB (*r* = −0.33, *p* < 0.01).

### 3.3. Mediation Model Analysis

This study first conducted regression analyses for the mediator variables and the dependent variable separately to establish the paths. Table 2 summarizes the key statistical indicators for each regression model.

1. Model 1 (Social Anxiety as Outcome): With Sports Learning Interest as the predictor, there was a significant negative predictive effect on Social Anxiety (*B* = −0.06, *t* = −13.21, *p* < 0.001). This suggests that students with higher interest in sports report lower levels of social anxiety.
2. Model 2 (Sleep Quality as Outcome): When Sports Learning Interest and Social Anxiety were entered as predictors, Sports Learning Interest significantly negatively predicted PSQI scores (*B* = −0.03, *t* = −9.47, *p* < 0.001), meaning it positively predicted sleep quality. Social Anxiety positively predicted PSQI scores (*B* = 0.20, *t* = 11.96, *p* < 0.001), indicating higher anxiety is associated with poorer sleep.
3. Model 3 (SWB as Outcome): When Sports Learning Interest, Social Anxiety, and Sleep Quality were simultaneously included, Sports Learning Interest showed a positive direct effect (*B* = 0.02, *t* = 8.58, *p* < 0.001). Social Anxiety (*B* = −0.08, *t* = −5.64, *p* < 0.001) and Sleep Quality (*B* = −0.47, *t* = −23.86, *p* < 0.001) were significant negative predictors (i.e., less anxiety and better sleep predict higher well-being).

These regression results suggest that Social Anxiety and Sleep Quality play partial mediating roles between Sports Learning Interest and SWB, as the direct effect of Interest on SWB remained significant but reduced.

Further analysis using Hayes PROCESS Model 6 verified the specific paths of the mediation effects (Table 3). The total indirect effect was significant (Effect = 0.18, 95% CI [0.15, 0.21]). Specifically, the mediation effect consisted of three significant pathways:

1. Path 1 (Ind 1): Sports Learning Interest → Social Anxiety → SWB (Effect = 0.03, 95% CI [0.02, 0.05]), accounting for 9.8% of the total effect.
2. Path 2 (Ind 2): Sports Learning Interest → Sleep Quality → SWB (Effect = 0.11, 95% CI [0.08, 0.13]), accounting for 29.9% of the total effect. This indicates that sleep is the most potent mediator.
3. Path 3 (Ind 3): Sports Learning Interest → Social Anxiety → Sleep Quality → SWB (Effect = 0.04, 95% CI [0.03, 0.05]), accounting for 11.3% of the total effect. This confirms the chain mediation hypothesis.

## 4. Discussion

This study systematically investigated the relationship between Sports Learning Interest and SWB in middle school students and verified the mediating mechanisms of Social Anxiety and Sleep Quality. The results show that Sports Learning Interest not only directly predicts SWB but also exerts indirect influence through the independent mediating roles of Social Anxiety and Sleep Quality, as well as the chain mediating role of both.

### 4.1. The Association Between Sports Learning Interest and SWB

This study found a significant positive correlation between Sports Learning Interest and SWB, validating H1 and consistent with previous evidence regarding PA promoting mental health (Ryan & Deci, 2017; Zhou & Wu, 2021).

When students are interested in sports, the process of participating in sports activities may help satisfy basic psychological needs, although the cross-sectional design of this study limits causal inference:

1. Competence: Gaining mastery over physical skills and body control strengthens self-efficacy.
2. Autonomy: Participating in activities one finds interesting fosters a sense of volition and agency.
3. Relatedness: Sports often involve shared goals and team collaboration, fostering connection (Standage et al., 2012).

The satisfaction of these psychological needs appears to be associated with SWB, though the directionality of this relationship warrants further investigation through longitudinal designs. Conversely, a lack of sports interest may lead to “amotivation,” resulting in social withdrawal, sedentary behavior, and lower life satisfaction (S. Lin et al., 2022). Therefore, cultivating Sports Learning Interest—rather than merely enforcing exercise duration—is an effective pathway to enhance adolescent SWB.

### 4.2. The Independent Mediating Roles of Social Anxiety and Sleep Quality

First, this study found support for the independent mediating role of Social Anxiety between Sports Learning Interest and SWB (H2). However, this finding should be interpreted with caution. It is possible that students with higher SWB are inherently more sociable and less anxious, rather than interest in sports directly reducing social anxiety. The cross-sectional design precludes ruling out such reverse causation. The physical education environment provides a social interaction context for adolescents that is distinct from the academic classroom. Sports Learning Interest drives students to engage, and this engagement creates opportunities for positive social feedback. As evidenced by experimental research (Luna et al., 2019), sport education models can improve emotional intelligence and reduce anxiety. Based on the relationship needs perspective of SDT, individuals with higher interest exhibit higher “social self-efficacy” in sports contexts. They are more focused on the activity than on self-presentation, which helps to reduce the fear of negative evaluation (Wu et al., 2025). Reduced levels of Social Anxiety improve the individual’s social adaptability and reduce the psychological cost of interpersonal interactions, thereby enhancing well-being.

Second, Sleep Quality demonstrated a significant mediating effect in the model (H3), with the largest indirect effect among the three pathways. This aligns with recent evidence linking PA, sleep, and psychological well-being (K. Li et al., 2025; Cheng et al., 2025). However, the magnitude of this mediating effect may be partly attributable to the operationalization of sleep quality as a unidimensional composite score. Given that the PSQI encompasses seven distinct components, future studies might benefit from examining which specific sleep dimensions (e.g., latency vs. efficiency) drive this mediation. This underscores that high-quality sleep is a pivotal physiological bridge. The results are consistent with empirical evidence in Asian contexts, where sleep problems (such as difficulty falling asleep due to academic pressure) are core physiological factors predicting declines in adolescent SWB (Otsuka et al., 2020). Sports Learning Interest promotes regular PA, which helps optimize biological rhythms and neuroendocrine balance (e.g., cortisol regulation), thereby improving sleep structure (Gruber et al., 2012). This study supports the logical link of “Exercise-Sleep-Well-being.” Notably, the impact of sleep quality on psychological distress may be moderated by personality traits; introverted individuals with poor sleep quality often face higher mental health risks (Zheng et al., 2026). Thus, improving sleep through sports interest is particularly important for such high-risk groups, as sports participation maintains SWB by safeguarding basic physiological health.

### 4.3. The Chain Mediating Pathway of Social Anxiety and Sleep Quality

A notable finding of this study is the identification of the chain influence path from Social Anxiety to Sleep Quality (H4). While this aligns with theoretical expectations and prior research (Alvaro et al., 2013; W.-H. Zhang et al., 2026), it is important to note that mediation analysis in cross-sectional data does not establish causality. The observed chain effect may reflect concurrent associations rather than sequential causal processes. This pathway is supported by clinical evidence; systematic reviews indicate that anxiety is a significant and robust predictor of sleep disorders (Alvaro et al., 2013). Simultaneously, this result supports the Cognitive Model of Insomnia (Harvey, 2002).

The mechanism can be understood as follows: Individuals with high Social Anxiety are prone to “pre-sleep cognitive hyperarousal.” They engage in ruminative thinking about social interactions, worrying about how they were perceived during the day or how they will perform tomorrow. This cognitive activity triggers physiological arousal (sympathetic activation), making it difficult to fall asleep or stay asleep.

If adolescents possess high Sports Learning Interest, they are likely to experience more positive social interactions and feel more competent during the day. This reduces the “fuel” for social anxiety. Consequently, with lower Social Anxiety, the tendency for pre-sleep rumination decreases, facilitating a quicker and more peaceful transition to sleep. Diary studies have also confirmed this reciprocal association between daytime emotional states and that night’s sleep quality (Kouros & El-Sheikh, 2015).

This chain pathway indicates that the enhancement of well-being by sports activity involves a continuous process from psychological adaptation to physiological function. Recent network analysis research has similarly found that sleep cognition and self-deprecation are core nodes connecting sleep problems and psychological problems (S. J. Wang et al., 2026). Although this study did not directly measure these micro-cognitive variables, the results suggest that Sports Learning Interest acts as a protective factor that blocks the transformation of social stress into physiological sleep problems. This finding aligns with and extends recent research on university students which proposed a chain mediation model involving anxiety and well-being (Zhu et al., 2025), by specifically isolating “Social Anxiety” as the key psychosocial stressor relevant to adolescents.

These findings extend SDT by illustrating that the satisfaction of intrinsic motivation, manifested as sports interest, relates to a potential buffering effect on psychosocial stressors such as Social Anxiety, which is further linked to physiological indicators including Sleep Quality. This provides a more integrated ‘psychological-to-physiological’ framework for understanding adolescent well-being within a correlational context.

### 4.4. Research Limitations and Future Prospects

This study has several limitations that should be acknowledged. First, the reliance on self-report measures introduces potential for recall bias and social desirability bias. Notably, constructs such as Sports Learning Interest and Social Anxiety were assessed using composite scores without rigorous measurement model validation beyond CFA fit indices. Future research should employ confirmatory factor analyses at the item level to verify factorial validity within the specific sample. Second, the cross-sectional design precludes causal inference, despite the theoretical model implying directional relationships. Although mediation analysis was applied, it cannot establish temporal precedence; longitudinal or experimental designs are needed to verify the proposed causal chain. Third, although we addressed concerns regarding construct validity by supplementing Confirmatory Factor Analysis (CFA) prior to hypothesis testing, the current study ultimately relied on composite scores using the PROCESS macro. This regression-based approach does not fully account for item-level measurement errors inherent in latent variables. Future research should prioritize a full Structural Equation Modeling (SEM) approach to simultaneously estimate measurement and structural parameters, thereby yielding more precise estimations of complex multidimensional constructs. Fourth, this study did not differentiate between types of sports (e.g., team vs. individual). Different sports contexts may confer distinct social and psychological benefits (Liu & Zhong, 2023), and future research should explore these differential impacts. Fifth, potential confounding variables such as personality traits, family background, and academic pressure were not controlled for; the moderate explanatory power of the model suggests that unmeasured variables may play important roles. Future research could employ smart wearable devices (e.g., actigraphy) to collect objective activity and sleep data and use longitudinal tracking to establish temporal ordering among the variables.

## 5. Conclusions

Through an empirical investigation of 1764 adolescents, this study examined the associations between Sports Learning Interest and SWB and explored potential mediating mechanisms involving Social Anxiety and Sleep Quality. The conclusions are as follows:

1. Direct Association: Sports Learning Interest was significantly and positively associated with the SWB of middle school students, even after accounting for Social Anxiety and Sleep Quality.
2. Indirect Effects: The impact is mediated through three pathways:
  - The independent mediating role of Social Anxiety;
  - The independent mediating role of Sleep Quality;
  - The Chain Mediating Role of Social Anxiety → Sleep Quality.

This conclusion reveals that the enhancement of adolescent well-being by Sports Learning Interest is a synergistic process from the inside out, coordinating mind and body. It suggests to educators that school physical education reform should shift from a “single skill orientation” to an “intrinsic motivation orientation.” By creating a supportive teaching environment that stimulates students’ sports interest, schools can build a mental health protective net that integrates social adaptation with physiological restoration.

## Figures and Tables

**Table 1:** Descriptive Statistics and Correlation Coefficients of Main Variables.

Variable	*M*	*SD*	1	2	3	4
Sleep Quality (PSQI)	5.03	3.00	1			
Sports Learning Interest	86.58	22.21	−0.30 **	1		
Social Anxiety	7.11	4.13	0.34 **	−0.30 **	1	
SWB	10.83	2.87	−0.58 **	0.35 **	−0.33 **	1

Note: *N* = 1764; ** *p* < 0.01. PSQI scores are reversed in interpretation (High Score = Low Quality).

**Table 2:** Regression Model Analysis Results.

Dependent Variable	Independent Variable	*R* ^2^	*F*	*B*	*t*	95% CI
Social Anxiety	Sports Learning Interest	0.09	174.53 ***	−0.06	−13.21 ***	[−0.06, −0.05]
Sleep Quality (PSQI)	Sports Learning Interest	0.16	165.18 ***	−0.03	−9.47 ***	[−0.04, −0.02]
	Social Anxiety			0.20	11.96 ***	[0.17, 0.23]
SWB	Sports Learning Interest	0.38	361.64 ***	0.02	8.58 ***	[0.02, 0.03]
	Social Anxiety			−0.08	−5.64 ***	[−0.11, −0.05]
	Sleep Quality (PSQI)			−0.47	−23.86 ***	[−0.50, −0.43]

Note: *** *p* < 0.001. All coefficients are unstandardized.

**Table 3:** Direct and Indirect Effects Analysis.

Path	Standardized Effect	% of Total Effect	Bootstrap Lower CI	Bootstrap Upper CI
Total Indirect Effect	0.18	51.1%	0.15	0.21
Sports Learning Interest → Social Anxiety → SWB	0.03	9.8%	0.02	0.05
Sports Learning Interest →Sleep Quality → SWB	0.11	29.9%	0.08	0.13
Sports Learning Interest → Social Anxiety → Sleep Quality → SWB	0.04	11.3%	0.03	0.05

## Data Availability

The data presented in this study are available on request from the corresponding author. The data are not publicly available due to ethical restrictions.
